# Notes and Notices

**Published:** 1891-05

**Authors:** 


### NOTES AND NOTICES.

THE COMMENCEMENT OF THE HOMŒOPATHIC MEDICAL COLLEGE, OF MISSOURI, took place on Thursday, March 12th, in Pickwick Theatre. The exercises were opened by the Rev. Dr. H. F. Deters invoking the Divine blessing. Mr. Charles Kunkel then gave one of his own compositions upon the piano-forte, which was followed by a song and the encore of the Amphion Quartette. The Rev. J. W. Ford, D. D., then delivered the address on behalf of the Faculty, entitled the "Victories of Defeat," which was beautiful, instructive, and sensible. Miss Agnes Gray gave a violin solo, after which W. A. Edmonds, A. M., M. D., conferred the degree of Doctor of Medicine upon the following: Jacob Smith, John Dryden, W. B. Young, E. A. Elfeld, A. Killmer, J. B. Julian, Dennis Lyons, H. A. Lott, W. W. Minick, C. F. Hitchcock, Frank Saitz, R. Y. Henry, Miss E. D. Wilcox, Miss Lina Rosat, Miss Lizzie Lovejoy. After the delivery of the diplomas, Miss Louise A. Peebles sang a song, entitled "My Darling," which was succeeded by the presentation of flowers. After Mr. Kunkel had given another piece upon the piano, the benediction was pronounced by Rev. Dr. Deters and the assembly dispersed.

The efficient committee upon arrangements was composed of Dr. James A. Campbell, Dr. I. D. Toulon, and Dr. L. C. McElwee, Secretary, 219 S. Jefferson Avenue, St. Louis.

Commencement of Cleveland Homœopathic Hospital College.—The commencement exercises were held in the association hall of the new Y. M. C. A. building, Tuesday evening, March 24th. The graduating class consisted of eight members, seven men and a girl, whose names were Arthur Eugene Chamberlain, Robert Sinclair Evelyn, Lucy Stone Hertzog, Thomas Fletcher Hogue, Thaddeus Lincoln Johnson, James D. McAfee, Justus Elden Rowland, and Augustus B. Smith.

The exercises opened with prayer, after which Miss Marie St. Urbain executed a piano solo, grand galop de concert, by Kellerer. Prof. J. C. Sanders, M. D., Dean of the College, presented his report of the work of the College for the past year and the high average and individual standing of the graduating class.

Thaddeus L. Johnson, one of the graduating class and also a member of the Hahnemann Society, which is an adjunct of the College, delivered the society address, selecting as his subject, "Battle Fields." His theme was the renowned victories of the eminent physicians of the past and the present in battling the ravages of disease. After he had made his bow a pleasant little incident occurred which made a ripple of amusement on the placid literary sea. A little black-eyed girl, some three or four years old, trudged down the aisle with a big bunch of red, yellow, and white roses clasped tightly in her fat little hand, and, espying the orator, exclaimed, "Here, Mr. Johnson," and gave him the flowers.

Miss St. Urbain then executed Godefroid's "Danse de Sylphes" very cleverly upon the harp.

Rev. Dr. George R. Leavitt, of Plymouth Church, rose and said: "By the courtesy of your Professors and the Board of Trustees, the pleasant duty of conferring your degrees devolves upon me. I had nearly said bachelors' degree when I suddenly remembered that the term would hardly be applicable to the one of your number who is a lady. In my profession the end is sometimes to be a doctor. In yours you must be doctors to begin with. I hope you will be good ones. The field of medicine never presented in times past such opportunities as it now does. You will not fulfill all your hopes nor all the hopes which your alma mater has for you, for what man ever did fulfill all the hopes of himself or his friends. But it is wholly probable that you will all achieve success, and that I wish you with my whole heart."

Diplomas were then conferred on the members of the graduating class by Dr. Leavitt, who also conferred on Drs. Edward A. Darby, Stanton L. Hall, Myron H. Parmelee, Frank Kraft, and W. P. Phillips the ad eundem degree and announced that the honorary degree had been conferred on Dr. Launcelot Younghusband, of Detroit.

Rev. Dr. S. P. Sprecher, the pastor of the Third Presbyterian Church, delivered the address of the evening at this point.

Dr. H. F. Biggar followed Rev. Dr. Sprecher, after which he conferred the fellowship of the Hahnemann Society degree upon Drs. B. W. Baker, DeForrest Baker, H. F. Biggar, Joseph T. Cook, C. D. Ellis, E. R. Eggleston, Julia C. Jump, R. B. Rush, John Kent Sanders, Jacob Schneider, W. E. Wells, George Edward Turrill, and M. D. Wilson.

After the benediction the audience repaired to the banquet in the Hollenden.

International Hahnemannian Association.—To the Members: Richfield Springs is right before us. The Bureau of Surgery is short. If it is to make a fair showing, members will have to write for it soon. Too many have the notion that only cases requiring mechanical assistance belong to this bureau. A great mistake! Every one is requested to contribute the history of some case cured with medicine that would have been condemned to the knive by the old school; or some case, necessarily operative, rendered more surely or quickly successful, and more comfortable, by homœopathic medicine. We should put many such cases upon record, and confound our enemies.

Reader, this is addressed to *you*. Please send the title of your paper at the earliest moment practicable. Edmund Carleton, M. D., *Chairman*,
53 West 45th Street, New York.

Other Errors in the Published "Proceedings of the International Hahnemannian Association for 1890."—After *rising*, on, amel.: Phos. on p. 261, there should be inserted all of p. 264, beginning with *eyeballs*, p. 265, p. 266, and p. 267 to and including *light*, from: Ast., Gin., Merc. P. 262, beginning with Accommodation, p. 263, should be carried over to p. 267, when it will be seen to make continuous reading with sense. Other careless proof-reading is to be seen throughout the volume, and the omission of dashes throughout the repertory of Asthenopia makes it less valuable than was intended. G. H. C.

Ice in the Sick-Room.—A saucerful of shaved ice may be preserved for twenty-four hours with the thermometer in the room at ninety degrees F., if the following precautions are observed: Put the saucer containing the ice in a soup plate, and cover it with another. Place the soup plates thus arranged on a good, heavy pillow, and cover it with another pillow, pressing the pillows so that the plates are completely embedded in them. An old jack-plane set deep is a most excellent thing with which to shave ice. It should be turned bottom upward, and the ice shoved backward and forward over the cutter.

